# Electrically conductive hybrid organic crystals as flexible optical waveguides

**DOI:** 10.1038/s41467-022-35432-w

**Published:** 2022-12-22

**Authors:** Xuesong Yang, Linfeng Lan, Xiuhong Pan, Xiaokong Liu, Yilong Song, Xueying Yang, Qingfeng Dong, Liang Li, Panče Naumov, Hongyu Zhang

**Affiliations:** 1 State Key Laboratory of Supramolecular Structure and Materials, College of Chemistry, Jilin University, Changchun, 130012 P. R. China; 2 Smart Materials Lab, New York University Abu Dhabi, PO Box 129188, Abu Dhabi, UAE; 3 Department of Sciences and Engineering, Sorbonne University Abu Dhabi, PO Box 38044, Abu Dhabi, UAE; 4 Molecular Design Institute, Department of Chemistry, New York University, 100 Washington Square East, New York, NY 10003 USA; 5 Research Center for Environment and Materials, Macedonian Academy of Sciences and Arts, Bul. Krste Misirkov 2, MK‒1000 Skopje, Macedonia

**Keywords:** Organic molecules in materials science, Electronic devices, Mechanical properties, Optical materials

## Abstract

Hybrid materials capitalize on the properties of individual materials to attain a specific combination of performance assets that is not available with the individual components alone. We describe a straightforward approach to preparation of sandwich-type hybrid dynamic materials that combine metals as electrically conductive components and polymers as bending, momentum-inducing components with flexible organic crystals as mechanically compliant and optically transducive medium. The resulting hybrid materials are conductive to both electricity and light, while they also respond to changes in temperature by deformation. Depending on the metal, their conductivity ranges from 7.9 to 21.0 S µm^‒1^. The elements respond rapidly to temperature by curling or uncurling in about 0.2 s, which in one typical case corresponds to exceedingly fast deformation and recovery rates of 2187.5° s^‒1^ and 1458.3° s^‒1^, respectively. In cyclic operation mode, their conductivity decreases less than 1% after 10,000 thermal cycles. The mechanothermal robustness and dual functionality favors these materials as candidates for a variety of applications in organic-based optics and electronics, and expands the prospects of application of organic crystals beyond the natural limits of their dynamic performance.

## Abstract (web summary)

The poor conductivity of organic crystals hinders their potential on applications in flexible electronics, wearable devices, and soft robotics. Here, Naumov et al. develop a hybrid organic crystal that shows enhanced electrical conductivity and fast mechanical deformation due to temperature change.

## Introduction

An ideal smart dynamic material is light in weight, resistant to mechanical damage, resilient to temperature and pressure, has high work density, and can operate over an indefinite number of cycles. While it is a challenge to have all these assets combined in a single material, one particular traditionally overlooked class of engineering materials, organic crystals, has been recently realized to comprise some of these prerequisites^1–13^. Compared to other soft materials, the organic crystals are endowed with ordered, dense structures and many of them normally retain elasticity across a very wide temperature range up to their melting point, a property which is desirable for applications in extreme thermal conditions^14^. The organic crystals, when of good quality, are also optically transparent and, depending on their absorption spectrum can readily transduce visible and/or near-infrared light as either passive or active (fluorescent) waveguides^15–19^. However, since the common organic crystals are not normally electrically conductive, extending their applications to conductive components in electrical circuitry is not a straightforward undertaking. Moreover, mechanical deformation of crystals such as bending or twisting induced by light or heat is normally slow for practical applications that require instantaneous response, for instance, in dynamic elements for electrical switching or microfluidics. Aimed at compensating for the slow response of deformable organic crystals while simultaneously trying to diversify the set of stimuli that can be used to control the crystals’ shape^20–23^, here we propose a universal approach to preparation of conductive hybrid organic crystals that relies on combining flexible organic crystals with polymers and metals. The polymers, being chemically diverse, are known to be responsive to a wide array of environments (heat, light, magnetic field, chemical agents)^24–29^, while they also are exceedingly mechanically flexible and compliant, and can be engineered into shapes that are compatible with actual devices^20,21,30^. Since, with rare exceptions^31–33^, the organic flexible crystals are normally not significantly electrically conductive, the conductivity is introduced by a layer of metal that is deposited and intimately adheres to the crystal. The resulting hybrid materials are not only optically transmissive and electronically conductive, but they also respond to changes in temperature, and thus they can be used for thermal switching of optical and/or electrical circuits. Combining electrical conductivity and optical waveguiding in the same material has not been reported prior to this work for the emerging class of organic crystals. In one of the plethora of applications that we envision for the hybrid materials described here, this work opens prospects for temperature control over the optical transduction process via resistive (Joule) heating. This could be the basis for a range of applications related to optical switching or spatial control of light output by heating-induced deformation of the hybrid crystals. The strategy expands significantly the range of applications of organic crystals as optical waveguides, even in extreme conditions where the temperature of the waveguide would be maintained constant to prevent the material from thermal failure or to ensure that light passes through a constant optical path.

## Results and discussion

### Preparation of the hybrid flexible crystals

**Fig. 1:**
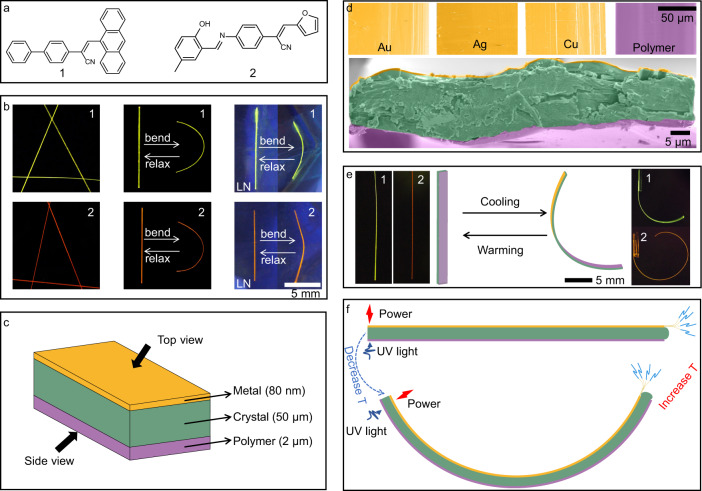
Preparation and structure of the hybrid conductive polymer organic crystals. **a** Chemical structures of the elastic crystals of 1 and 2. **b** Photographs of crystals of 1 and 2 in their straight state (left) and in the bent state after bending by squeezing at room temperature (middle) and at liquid nitrogen temperature (LN) (right). For improved contrast, the crystals were observed via their emission under 365 nm light against black background. **c** A schematic diagram showing the ‘sandwich’ M//1,2//P structure. **d** False-colored Scanning Electron Microscopy (SEM) images showing the surface and cross-sections of crystals M//1,2//P. The crystal is shown in green, the conductive metal layer is in orange, and the polymer coating is in purple color. **e** Deformation of the hybrid crystals M//1,2//P with change in temperature. **f** Illustration showing the concept of operation of a hybrid organic crystal for temperature-dependent dual transmission of both light and electricity.

The approach proposed here relies on the ability of many organic crystals to bend elastically, whereby they effectively transduce the bending moment through their ordered crystalline structures and can be cycled between their original straight shape and a deformed state practically indefinitely^34–36^. Organic crystals that are electrically conductive are rare. In this work, we propose a method that could be generalized and, in principle, could be applied to convert any non-conductive elastic organic crystal into an electrically conductive hybrid element for incorporation in flexible electronic devices. This is accomplished by coating organic crystals with a metal layer, which is inherently conductive to electricity. The approach is demonstrated, as a proof-of-concept, on crystals of two non-conductive elastic crystalline compounds, 1 and 2 (Fig. 1a, Supplementary Figs. 1 and 2). Centimeter-long slender crystals of these compounds were found to be elastic (Supplementary Table 1)^20^. While their crystals are straight at both ambient and low temperatures, they can be easily bent into a U-shape by application of an external force, and recover their straight shape immediately after the force has been removed (Fig. 1b). Due to the absence of low-temperature phase transitions, the crystals of 1 and 2 remain elastic even at cryogenic conditions, at least down to 77 K. Single-crystal X-ray diffraction analysis was performed on one crystal of each 1 and 2 at both 298 and at 100 K. As shown in Supplementary Figs. 3 and 4, the lengths of the unit cell axes are shortened (−0.71% ~ −1.40% for 1 and −0.92% ~ −1.01% for 2) upon cooling from room temperature. In addition, the molecular conformations of individual molecules at 100 K in 1 and 2 are only slightly changed compared with those at 298 K. As a result, the overall molecular packing structure and the directionality of the intermolecular interactions are retained at low temperature, which is reflected as retention of the capability for bending without fracture in cryogenic conditions. Moreover, the slightly stronger π⋅⋅⋅π interactions at low temperature might further enhance the tolerance of the structure to tensile forces/pressure. All of these factors contribute to the favorable elasticity of the crystals of 1 and 2 at low temperatures, as demonstrated by previous studies on other organic crystals, which are naturally devoid of glass transition and are thus thermoelastically superior to polymers^14,37^. This property stands as an advantage of these and other organic crystals over some materials such as elastomers, which normally undergo glass transition and become brittle at low temperatures^14^.

In the first step of the preparation of the hybrids, a layer of metal was deposited by vacuum thermal vapor deposition on one of the wide facets of the crystals. The roughness of the crystal surface was pronounced (Supplementary Fig. 5). The metal adhesion, similar to previous reports on the interactions across interfaces^38–41^, is probably due to van der Waals forces. In the second step, poly(urethane urea) (IPDI-SPU; for details, see the Methods section)^42^ was dissolved in ethanol and, by using a needle tip, it was cast as tiny droplets that spread uniformly across the opposite facet of the crystal. As the solvent evaporated at room temperature, a polymer film formed on that crystal surface. The adhesion between the polymer and the crystal involved stronger intermolecular interactions, such as hydrogen bonds^43^. The ensuing “sandwich”-type trilayer structures M//1,2//P (M = Au, Ag or Cu; P = IPDI-SPU) effectively combine the conductivity of the metal and elastomeric properties of the polymer with the elasticity of the organic crystal (Fig. 1c). The thickness of the polymer layer was typically about 2 µm, while that of the metal was kept about 80 nm to avoid significant stiffening of the crystal (Fig. 1d, Supplementary Fig. 6). The intimate contact between the polymer and the crystal was expected to result in a thermally responsive bilayer structure that would responds to thermal change by bending (Fig. 1e). Conversely, a crystal of another, chemically very different compound, 3 (Supplementary Fig. 7a), was found to be flexible at room temperature, but is brittle at low temperatures (Supplementary Fig. 7b‒d; Supplementary Movie 1)^8^. When the crystal surface is coated with polymer and the resulting hybrid crystal is placed in liquid nitrogen, it fractures (Supplementary Fig. 7d, Supplementary Movie 2). This result confirms that the elasticity of the hybrid materials at low temperature is primarily determined by the inherent elasticity of the crystal, rather than that of the polymer. Since only one side of the hybrid organic crystal was coated by the metal, the other accessible facet of the crystal remains optically translucent and, therefore, conducive to transmission of visible light (Fig. 1f). We note that this approach to hybrid structures is not limited as to the chemical composition of the organic crystal or the nature of the metal, and therefore an array of related hybrid sandwich-type structures can be prepared by combining different crystals, polymers and metals with a range of mechanical, electrical and optical properties.

### Conductivity of the hybrid flexible crystals

**Fig. 2:**
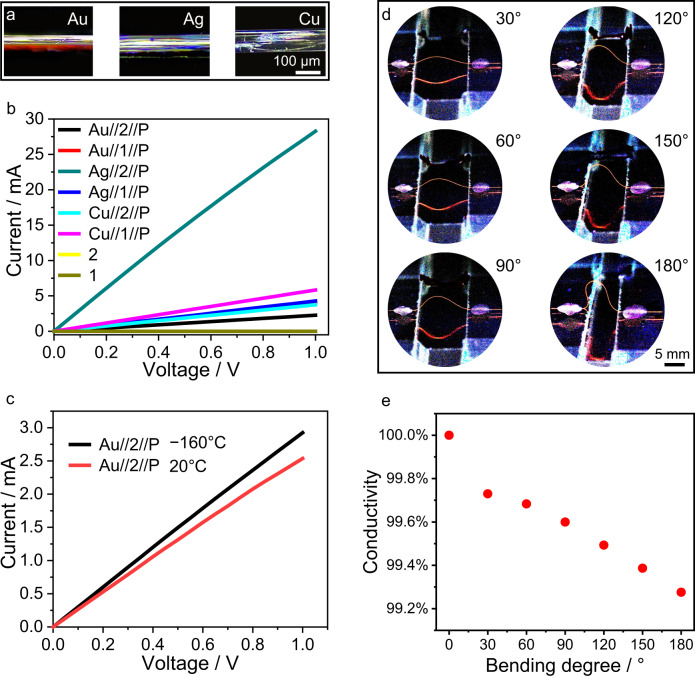
Electrical conductivity of the hybrid organic crystals. **a** Optical images of the hybrid organic crystals with different metal coatings (M = Au, Ag, Cu) (top view). **b** *I*‒*V* plot of crystals 1, 2 and M//1,2//P. **c** *I*‒*V* plot of Au//2//P at different temperatures. **d** Optical images of Au//2//P bent to different angles. The crystal is seen as red line between the two approaching surfaces. **e** Relative change in conductivity of Au//2//P at different bending angles.

Measurements of the electrical conductivity confirmed that while the native organic crystals of 1 and 2 are insulators, the metallic layer renders the hybrid structures electrically conductive (Fig. 2a). The conductivity, depending on the metal, ranged from 7.9 S µm^−1^ for Au//1//P to 21.0 S µm^‒1^ for Ag//2//P (Fig. 2b, Supplementary Table 2). The structures were stable upon conduction of electricity over prolonged periods of time (110 s, 10 mA; Supplementary Fig. 8) and at various temperatures. For example, the conductivity of Au//2//P was 7.9 S µm^‒1^ at 20 °C and 9.0 S µm^‒1^ at −160 °C (Fig. 2c, Supplementary Table 2). The mechanical robustness of the structures was assessed by in situ measurement of the conductivity of a crystal of Au//2//P while it was being bent at 20 °C (Fig. 2d). The crystal conductivity changed for only 0.72% when the crystal was bent at an angle of 180° compared to its straight state before bending (Fig. 2e, Supplementary Table 2). Moreover, we confirmed that the crystals of Au,Ag,Cu//1,2 can be prepared repeatedly, and the conductivity between different samples is quite consistent, demonstrating the reproducibility of the preparation method (detailed data on a large number of samples is provided in Supplementary Table 3, Supplementary Figs. 9–11). This retention of conductivity indicates the potential of these hybrid materials as candidates for flexible conductive components in electrical circuits^31,33^.

### Application of the hybrid flexible crystals for dual transmission of both light and electricity

**Fig. 3:**
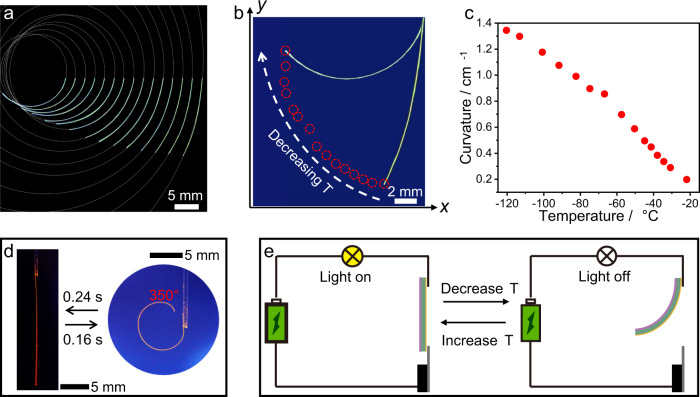
Bending of hybrid organic crystals. **a** Overlapped optical images of Au//1//P at different angles upon bending induced by cooling. **b** Tracking of the position of the free end of Au//1//P in **a** at different temperatures. **c** Dependence of the curvature of Au//1//P on temperature. **d** Instant response of Au//2//P with cooling and heating. **e** Schematic diagram of thermal switching of an electrical circuit using the hybrid switching element.

Inspection of the hybrid materials showed that while the metal layer is thin and granular (Fig. 1c, d, Supplementary Fig. 6), the polymer layer is compact and adheres firmly to the crystal. The differential thermal expansion between the polymer and the crystal was expected to generate a bending moment upon temperature change, as it has been demonstrated with other crystal-polymer hybrids^20,21^. As shown in Supplementary Fig. 12, the polymer coating provides elasticity and ensures that the crystals are bendable at low temperature, thereby significantly extending the operational temperature range of the hybrid crystals. Indeed, cooling of Au//1//P in air between −22 °C and −121 °C (the temperature was determined by direct measurement) induces visible bending of the hybrid element (Fig. 3a). Optical images showing the various degree of bending of Au//1//P are overlapped in Fig. 3a, the tracked free end of sample while being cooled is shown in Fig. 3b, and the bending curvature, plotted as a function of temperature is shown in Fig. 3c. Upon transfer of the hybrid crystals from ambient environment (20 °C) to LN (–160 °C) they bend rapidly. For example, upon flash-cooling the Au//2//P curled in 0.16 s, and after it was removed from the liquid nitrogen it recovered its original shape in 0.24 s. These times required for deformation correspond to angular bending rate of 2187.5° s^‒1^ and straightening rate of 1458.3° s^‒1^ (Fig. 3d, Supplementary Movie 3). These results indicate that the dynamic elements respond very fast to temperature change, and this rapid response is a significant advantage over the (uncoated) elastic crystals, which are known to have slow response to light^35^. The strong bending of the hybrid structures can be used to design simple temperature-switching devices where the coated crystal, integrated into an electrical circuit, makes contact with a conductive plate with its free end. One such proof-of-concept design is shown in Fig. 3e, where the crystal can be thermally bent, whereupon it breaks the circuit and acts as a thermal electrical fuse. The flow of electric current can be reinstated by uncurling of the crystal. This property was an inspiration to construct a simple temperature-responsive device, which is shown in Supplementary Fig. 13a.

**Fig. 4:**
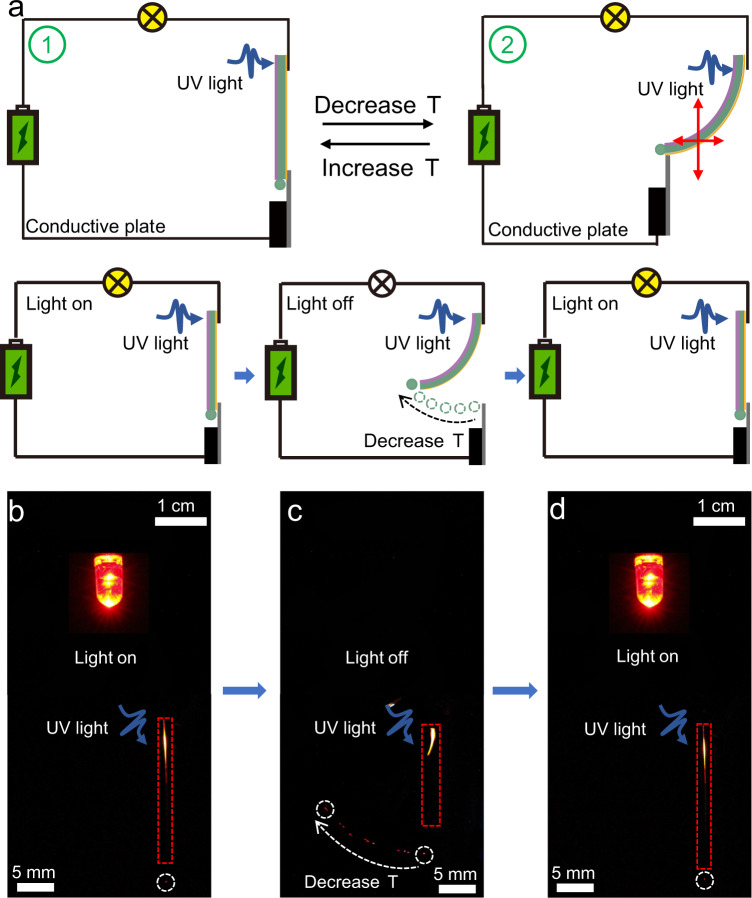
Hybrid organic crystals as optical and electrical switches for dual signal transmission. **a** Schematic diagram of optical/electrical dual signal temperature switching of an electrical circuit at ambient temperature (1) and upon cooling (2). The position of the free end of the bent crystal depends on the temperature, and the conductive plate assures current flow (The optical signal transmission is represented by the green circle). **b**‒**d** Schematic of an optical/electrical dual signal transmission circuit and image of the actual setup with Au//2//P. The bulb was used as an indicator. The crystal was excited by a 355 nm laser light. The position of waveguide output reflects the environmental temperature (marked with a white dashed line). The white arrow indicates the change of the output upon cooling. The crystals are highlighted with rectangular red contours.

One of the most important recent developments in the materials science of organic crystals is the focus on the potential they hold for light-weight, defect-free, and low-loss flexible optical waveguides. Organic crystals can transduce photons both at macro- and micro-scale, as well as in the visible and near-infrared regions^44–48^. One of the main goals in this line of pursuit has been to broaden the range of stimuli that can be used for spatiotemporal control over the optical output^20,49^. Another recent advancement in this burgeoning field has been directed towards expanding the range of applications of these waveguides to extreme conditions, and specifically, for application in cryogenic environments^21,37^. The hybrid elements reported here are conducive to visible light in both straight and bent states, as shown by crystals irradiated at 355 nm in Supplementary Fig. 14a‒d. The decrease in light intensity at the crystal end with increasing optical path length (Supplementary Fig. 14e‒h) was used to calculate the optical loss coefficients by following a standard procedure^50^. In the straight and bent states, the optical loss coefficients were found to be 0.15466 dB mm^‒1^ and 0.15913 dB mm^‒1^ for Au//1//P, and 0.42887 dB mm^‒1^ and 0.43403 dB mm^‒1^ for Au//2//P (Supplementary Fig. 14i‒l). Since the hybrid crystal is electrically conductive and optically transmissive, the direction of light output through the crystal can be controlled while the crystal simultaneously acts as an electrical switch (Fig. 4a‒d). As demonstrated in Fig. 3b, the position of the free end of the crystal is determined by the temperature, and this property enables switching on/off of a circuit at a specific temperature simply by adjusting the position of the conductive plates (Fig. 4a, Supplementary Fig. 13b).

### Durability of the hybrid flexible crystals

**Fig. 5:**
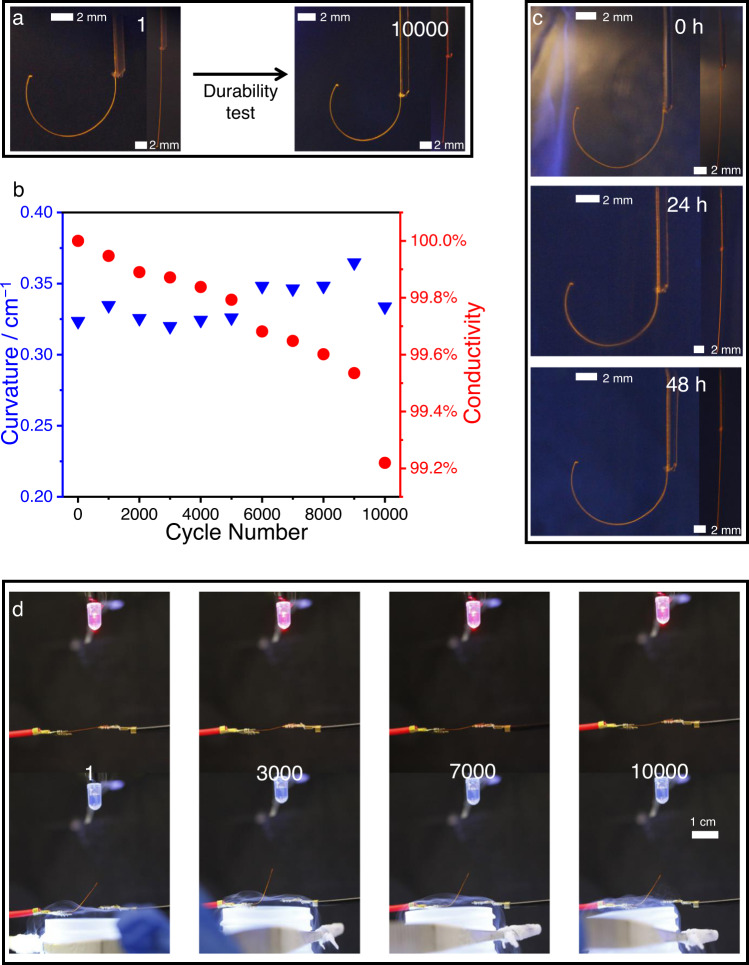
Durability upon cyclic operation. **a** Optical images of Au//2//P after the first and 10,000th cycle of bending. **b** Curvature and conductivity change of Au//2//P during cyclic operation. **c** Optical images of Au//2//P placed in a liquid nitrogen for 0, 24 and 48 h. **d** Optical images of Au//2//P and the experimental setup used to perform the durability test.

One example of a device that performs this task is shown in Figs. 3e and 4a. The robustness of the device over prolonged cyclic operation is one of the critical factors to be assessed in view of practical applications. As shown in Supplementary Figs. 15 and 16, the hybrid element can conduct electricity at least 24 h without interruption. The change in conductivity of M//2 (M = Au, Ag, Cu) after a long period of time was tested separately. As shown in Supplementary Fig. 17, the conductivity of M//2 remained almost unchanged after 40 days; it decreased for only 1.87% for Au//2, 0.32% for Ag//2, and 0.25% for Cu//2 relative to the initial values (Supplementary Table 4). The current and voltage were also stable over time (Supplementary Fig. 18). To assess the cyclability, a crystal was cycled between 20 °C and −160 °C, and photographed after each cycle (Fig. 5a; Supplementary Fig. 19). As shown in Fig. 5b, the bending capability of Au//2//P showed fluctuations in the durability test, however without significant decrease in performance. The crystals maintained their elasticity and were able to bend and straighten up after 48 h in liquid nitrogen (Fig. 5c). The cycling of the switching properties was tested in thermally variable environment by approaching to and retraction from liquid nitrogen of the hybrid element (Fig. 5d). Surprisingly, the crystal was very robust and was able to switch the electrical circle even after 10,000 thermal cycles. As shown in Fig. 5b, the conductivity showed slight decrease of only 0.78% after 10,000 cycles of bending and straightening (Supplementary Fig. 20 and Supplementary Table 4). It was suspected that one of the possible reasons for the decrease of efficiency could be partial detachment of some metal particles from the crystal. However, inspection of the SEM images did not show apparent changes of the surface or loss of the metal coating (Supplementary Fig. 21), and therefore we attribute this decrease to interparticle surface defects in the metallic layer.

In summary, we report a simple and universally applicable method for preparation of sandwich-type thermally and mechanically robust conductive flexible structures. The structures are concomitantly transducive to both light and electricity, and remain elastic at low temperatures. The approach described here does not pose any limitations as to the chemical nature of the elastic crystal, the metal and the adhesive polymer, and we demonstrate that different crystals can be used in combination with different metals. The electrical conductivity of silver-coated crystals was found to be particularly high; it can reach up to 21.0 S µm^‒1^ while the material remains flexible even at low temperature. The hybrid organic crystals are exceedingly mechanically robust; they respond instantly and repeatedly to low temperatures. This approach extends the potential of flexible organic crystals in applications such as low-temperature detectors, information transmission, and flexible optoelectronics, among others.

## Methods

### Materials

All the solvents and starting materials for syntheses were purchased from commercial resources and used as received without further purification. The steps for the synthesis of compounds 1 and 2 can be found in Supplementary Figs. 1, 2. Compound 2 was synthesized according to literature procedure^20^ (Supplementary Figs. 22–25), and compound 3 was purchased from Ennegy Inc. Dichloromethane solutions of compounds 1–3 were placed in test tubes, and approximately triple volume of ethanol was added along the wall of each test tube without disturbing the solution. After 1 or 2 weeks, at room temperature, needle-like crystals 1–3 were obtained.

### Metal deposition on crystal surface

The metal was placed in a molybdenum evaporation boat in a vacuum thermal evaporator, and the crystal was placed in a sample tray above the metal boat. The interior was evacuated to 4 × 10^‒4^ Pa by using a molecular pump. The temperature was controlled by controlling the current of the evaporation boat, so that the metal melt and boiled, and the metal vapor gradually condensed on the sample to form a metal film. The coating speed was maintained at 0.6‒0.8 Å s^‒1^, which resulted in deposition of about 80 nm of metal onto the crystal surface.

### Fabrication of hybrid flexible crystals Au,Ag,Cu//1,2//P

A layer of metal was first deposited on one broad side of the crystal by vacuum thermal vapor deposition. Subsequently, IPDI-SPU (IDPI: alicyclic hexatomic isophorone diisocyanate, SPU: supra-poly(urethane-urea)) was dissolved in ethanol at a concentration of (0.1 g/mL), and by using a needle tip it was cast as tiny droplets that spread uniformly across the opposite facet of the crystal. As the solvent evaporated at room temperature, a polymer film formed on the surface of this crystal.

### X-ray crystallographic analysis

The single-crystal X-ray diffraction data was collected on a Rigaku Synergy-DS Diffractometer. The data collection, integration, scaling, and absorption corrections were performed by using the Bruker Apex 3 software^51^. The structures were solved with direct methods by using Olex2^52^ and refined with the full-matrix least-squares method on *F*^2^. The non-hydrogen atoms were refined anisotropically. The positions of the hydrogen atoms were calculated and refined isotropically. The program PLATON was used for the geometrical calculations^53^. The graphics related to the structures were generated with Mercury 4.2.0^54^. Additional details crystallographic data are provided in Supplementary Table 5.

## Supplementary information

Supplementary Information

Description of Additional Supplementary Files

Supplementary Video 1

Supplementary Video 2

Supplementary Video 3

## Data Availability

Crystallographic data for the structures reported in this Article have been deposited at the Cambridge Crystallographic Data Centre, under deposition number CCDC 2157104. These data can be obtained free of charge from The Cambridge Crystallographic Data Centre via www.ccdc.cam.ac.uk/data_request/cif. All data are available from the corresponding authors upon request.
