# Supplementary material for: Electrically conductive hybrid organic crystals as flexible optical waveguides
Source: Nat Commun. 2022 Dec 22;13:7874. doi: 10.1038/s41467-022-35432-w (PMC9780324; doi:10.1038/s41467-022-35432-w)
Supplement: Supplementary file 1 — Supplementary Information [file 41467_2022_35432_MOESM1_ESM.pdf]

Supplementary Information

Electrically Conductive Hybrid Organic Crystals as Flexible Optical Waveguides

Xuesong Yang,[1] Linfeng Lan,[1] Xiuhong Pan,[1] Xiaokong Liu,[1] Yilong Song,[1] Xueying Yang,[1] Qingfeng Dong,[1] Liang Li,[2,3]* Panče Naumov,[2,4,5]* and Hongyu Zhang[1]*

[1]State Key Laboratory of Supramolecular Structure and Materials, College of Chemistry, Jilin University, Changchun 130012, P. R. China

[2]Smart Materials Lab, New York University Abu Dhabi, PO Box 129188, Abu Dhabi, UAE

[3]Department of Sciences and Engineering, Sorbonne University Abu Dhabi, PO Box 38044, Abu Dhabi, UAE

[4]Molecular Design Institute, Department of Chemistry, New York University, 100 Washington Square East, New York, NY 10003, USA

[5]Research Center for Environment and Materials, Macedonian Academy of Sciences and Arts, Bul. Krste Misirkov 2, MK–1000 Skopje, Macedonia

*Corresponding authors. Emails for correspondence: liang.li@sorbonne.ae (L. L.); pance.naumov@nyu.edu (P. N.); hongyuzhang@jlu.edu.cn (H. Z.)

Supplementary Methods

General information

^{1}H and $^{13}C\{^{1}H\}$ NMR spectra were recorded on an Agar Scientifica 400 MHz or a Bruker Avance or 500 MHz spectrometers with tetramethylsilane as internal standard. The mass spectra were recorded on a Thermo Fisher ITQ1100 mass spectrometer. The elemental analyses were performed on an Elementar Vario Micro Cube analyzer. The scanning electron microscopy (SEM) images were obtained on the FEI Quanta 450 operated at 5–10 kV. The emission spectra were recorded on a Maya2000 Pro CCD spectrometer. For the optical waveguiding tests the crystals were irradiated by the third harmonic (355 nm) of an Nd: YAG (yttrium-aluminum-garnet) laser at a repetition rate of 10 Hz and a pulse duration of about 10 ns. The energy of the laser was adjusted by using the calibrated neutral density filters. The beam was focused on a stripe whose shape was adjusted to 3.3 × 0.6 mm by using a cylindrical lens and a slit. The edge emission spectra were recorded on a Maya2000 Pro CCD spectrometer. The I–V curves were measured by a Keithley 2400 source meter. The deposition of metals on the crystal surface was carried out by vacuum thermal vapor deposition (QHV-R20). The conductivity of the hybrid organic crystals was determined by voltammetry based on the Eq. 1:

$$\sigma = (I \times L) / (U \times S) \qquad (1)$$

where: σ is the conductivity of the hybrid organic crystal/($S\cdot m^{-1}$), U is the voltage/V; I is the current/A; S is the cross-sectional area of the hybrid organic crystal/m^2; L is the distance between the two electrodes of the hybrid organic crystal/m.

Supplementary Figures

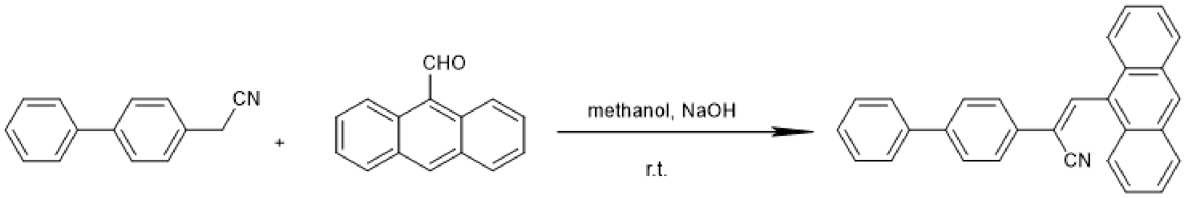

Supplementary Figure 1. Synthetic procedure for preparation of compound 1. 4-Biphenylacetonitrile (1.93, 10 mmol) and 9-anthraldehyde (2.06 g, 10 mmol) were added to ethanol (50 mL). NaOH (0.40 g, 10 mmol) was then added, and the mixture was stirred for 4 h at room temperature. The mixture was filtered to have a yellow solid, which was dissolved in dichloromethane and washed with brine. After drying over Na_2SO_4, the solvent was removed by vacuum roto-evaporation. The resulting crude product was purified by column chromatography using dichloromethane as an eluent to obtain compound 1 (3.10 g, 78%) as a yellow powder. ^{1}H NMR (Chloroform-*d*, 500 MHz) δ 9.03 (s, 1H), 8.77 (s, 1H), 8.24 – 8.17 (m, 2H), 8.16 – 8.06 (m, 4H), 7.95 – 7.90 (m, 2H), 7.83 – 7.77 (m, 2H), 7.62 (qd, J = 6.7, 3.4 Hz, 4H), 7.54 (t, J = 7.7 Hz, 2H), 7.48 – 7.41 (m, 1H). ^{13}C NMR (Chloroform-*d*, 126 MHz) δ 142.64, 139.90, 139.76, 132.12, 131.25, 129.44, 129.09, 128.99, 127.98, 127.84(2C), 127.10, 126.65, 126.54, 125.51(2C), 125.02, 120.83, 116.50. MS (ESI+): m/z calcd for $C_{29}H_{19}N$ $[M+H]^+$: 381.15; Found: 381.31. Anal. calcd (%) for $C_{29}H_{19}N$: C, 91.31; H, 5.02; N, 3.67. Found: C, 91.38; H, 5.07; N, 3.60.

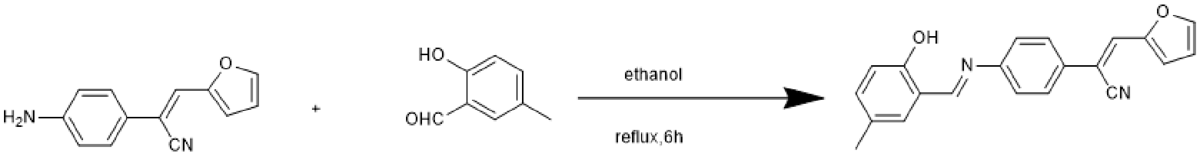

Supplementary Figure 2. The synthetic procedure of compound 2. 2-(4-aminophenyl)-3-(furan-2-yl)acrylonitrile (2.10 g, 10 mmol) and 2-hydroxy-5-methylbenzaldehyde (1.36 g, 10 mmol) were dissolved in ethanol (50 mL). After refluxing for 6 h, the resultant mixture was cooled down to room temperature, and was filtered and washed with ethanol. The crude product was purified by column chromatography using dichloromethane and petroleum ether (V/V= 4:1) as the eluent to produce compound 2 as an orange-red solid (3.02 g, 92.1% yield). ^{1}H NMR (DMSO-d_6, 400 MHz) δ12.64 (1H, s), 8.95 (1H, s), 8.03 (1H, d, J = 1.8 Hz), 7.94 (1H, s), 7.81 (2H, d, J = 8.5 Hz), 7.53 (2H, d, J = 8.5 Hz), 7.47 (1H, d, J = 2.2 Hz), 7.25 (1H, dd, J = 8.4, 2.2 Hz), 7.17 (1H, d, J = 3.5 Hz), 6.89 (1H, d, J = 8.4 Hz), 6.78 (1H, dd, J = 3.6, 1.8 Hz), 2.28 (3H, s). ^{13}C NMR (DMSO-d_6, 101 MHz) δ 164.11, 158.68, 150.24, 149.13, 146.91, 134.86, 132.79, 132.19, 128.55, 128.31, 127.01, 122.74, 119.52, 118.07, 117.69, 117.02, 113.76, 105.57, 20.45. MS (ESI+): m/z calcd for $C_{21}H_{16}N_2O_2$ $[M+H]^+$: 328.12; Found: 328.07. Anal. calcd (%) for $C_{21}H_{16}N_2O_2$: C, 76.81; H, 4.91; N, 8.53. Found: C, 76.96; H, 4.98; N, 8.67.

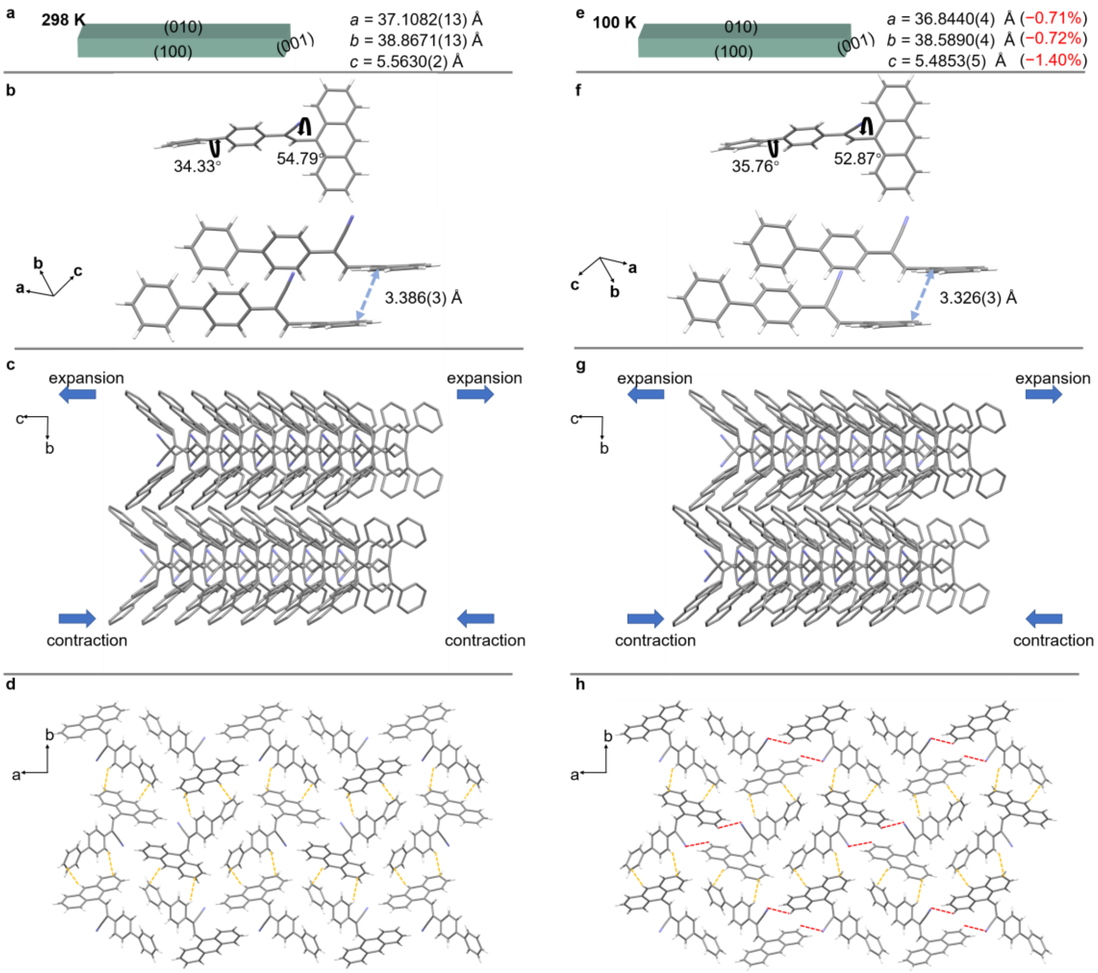

Supplementary Figure 3. Molecular and packing features of the needle-shaped crystal of 1 at 298 K and 100 K elucidated by X-ray diffraction analysis. (a) Schematic presentation of a crystal of 1 at 298 K with face indices. (b) Structure of 1 in the single crystal with the key torsion angles and the distance of the π···π interactions shown (a view perpendicular and along the plane defined by the central benzene ring). (c) The purported expansion and contraction directions of outer and inner arcs that is likely to occur during the bending process, respectively. (d) Molecular packing viewed along the c axis (the yellow dotted lines represent C—H···π interactions). (e) Schematic presentation of the crystal of 1 at 100 K with face indices. (f) Structure of 1 in the single crystal with the key torsion angles and distance of the π···π shown (a view perpendicular and along the plane defined by the central benzene ring). (g) The purported expansion and contraction directions of outer and inner arcs that are likely to occur during the bending process, respectively. (h) Molecular packing viewed along the c axis (the yellow dotted lines represent C—H···π interactions, the red dotted lines represent C—H···N hydrogen bonds).

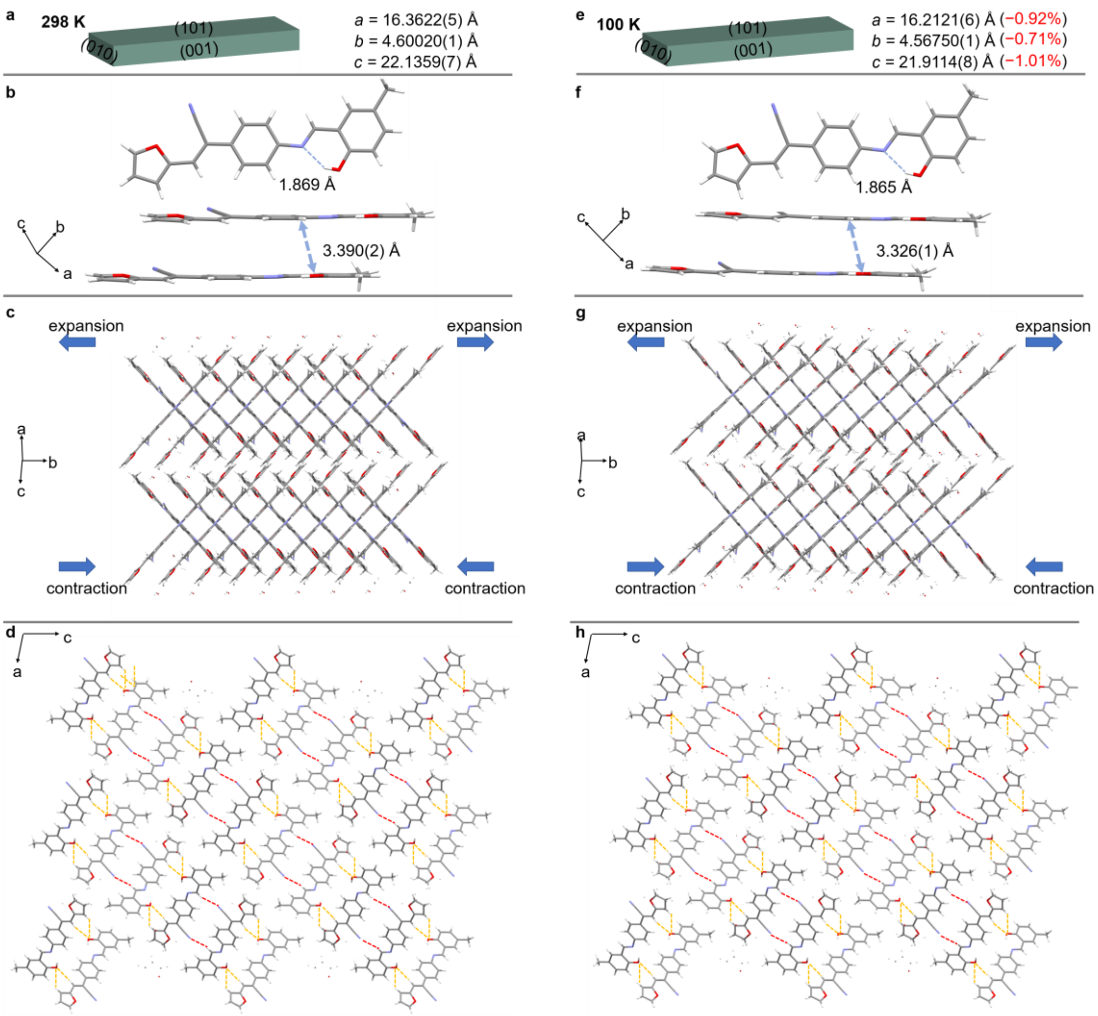

Supplementary Figure 4. Molecular and packing features of the needle-shaped crystal of 2 at 298 K and 100 K. (a) Schematic presentation of the crystal of 2 at 298 K with face indices. (b) Structure of 2 in the single crystal with the key torsion angles (the dashed blue line represents the intramolecular hydrogen bond) and distance of the π···π interactions (a view perpendicular and along the plane defined by the central benzene ring) shown. (c) The expansion and contraction directions of outer and inner arcs formed in the bending process, respectively. (d) Molecular packing of crystal 2 viewed along the b axis (the yellow dotted lines represent C—H···N hydrogen bonds and the red dotted lines represent the C—H···O hydrogen bonds). (e) Schematic representation of the crystal at 100 K with face indices. (f) Structure of 2 in the single crystal with the key torsion angles (the dashed blue line represents the intramolecular hydrogen bond) and the distance of the π···π interactions (a view perpendicular and along the plane defined by the central benzene ring) shown. (g) The purported expansion and contraction directions of outer and inner arcs that probably occurs during the bending process, respectively. (h) Molecular packing viewed along the b axis (yellow dotted lines represent C—H···N hydrogen bonds and the red dotted lines represent C—H···O hydrogen bonds).

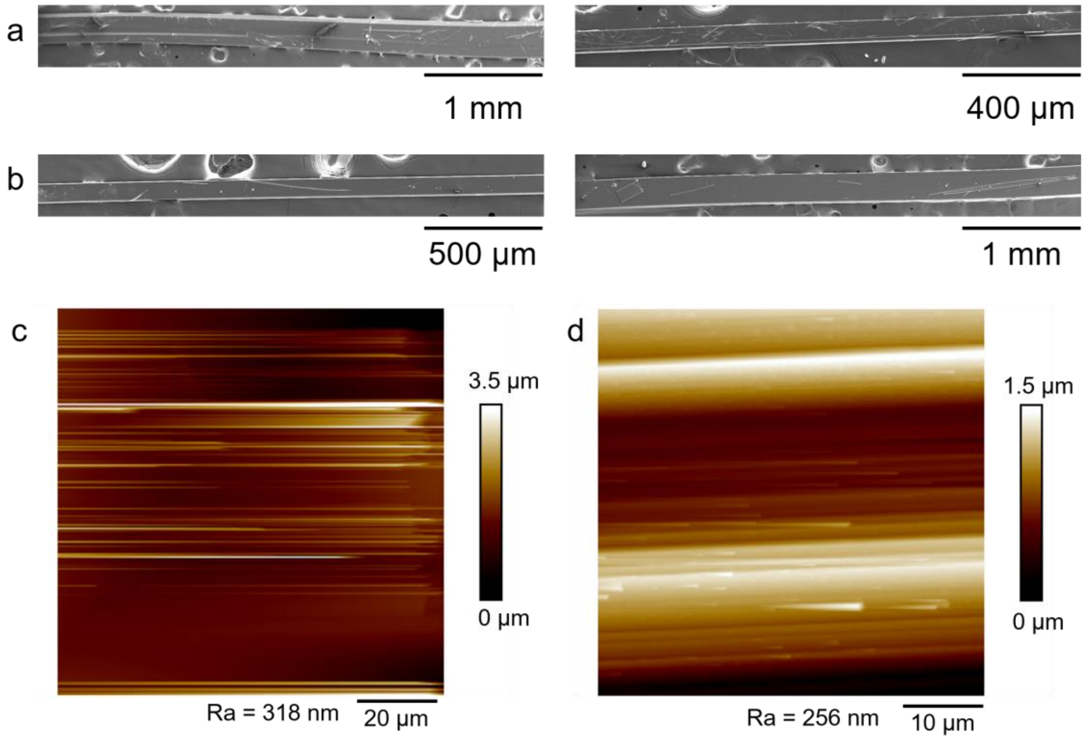

Supplementary Figure 5. The SEM and AFM testing of hybrid crystals. SEM pictures of the surfaces of crystals 1 (a) and 2 (b). AFM images of the surfaces of crystals 1 (c) and 2 (d).

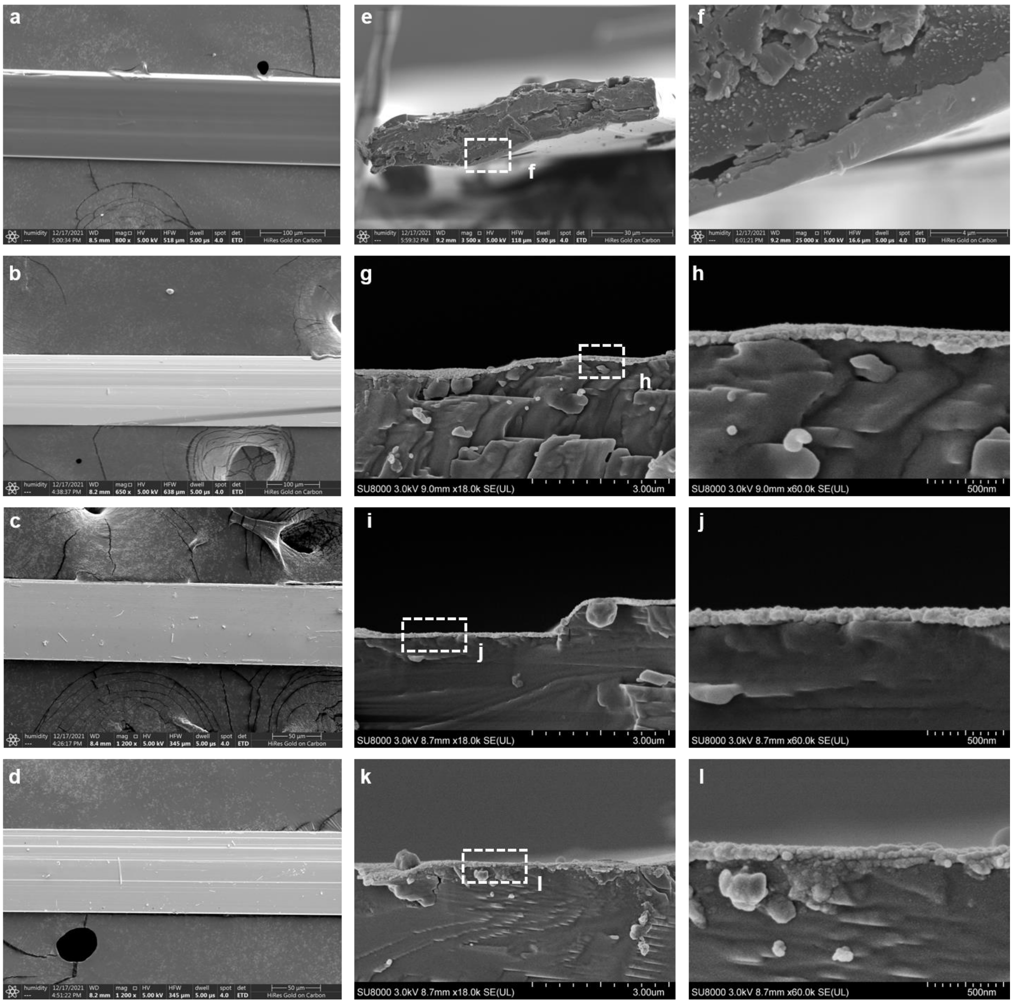

Supplementary Figure 6. SEM images of the samples. (a–d) Surface of polymer and Au, Ag, Cu //2//P, (a) polymer, (b) Au//2//P, (c) Ag//2//P, (d) Cu//2//P. (e–l) cross-section of polymer and Au, Ag, Cu //2//P, (e–f) polymer, (g-h) Au//2//P, (i–j) Ag//2//P, (k-l) Cu//2//P.

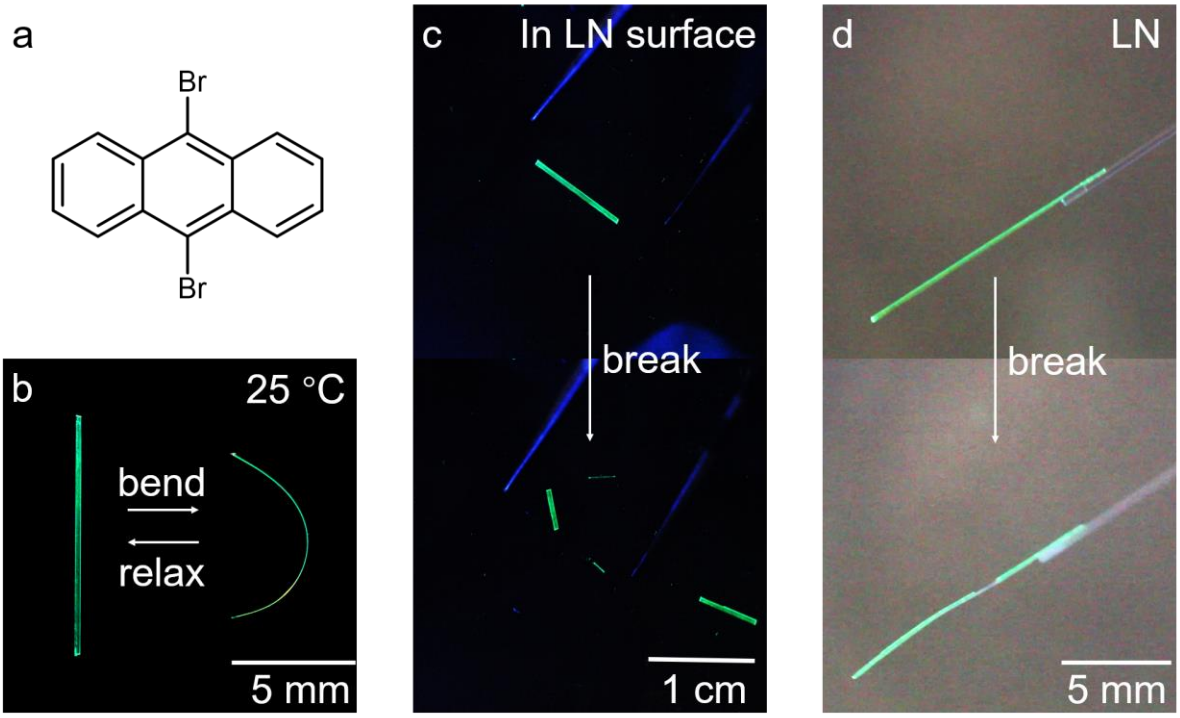

Supplementary Figure 7. Mechanical properties of a crystal of compound 3. (a) Chemical structure of 3. (b) Photograph of crystal 3 bent at room temperature. (c) Photograph of a crystal of 3 that has disintegrated at low temperature. (d) Photograph of a *hybrid* organic crystal made of 3 coated with polymer that has fragmented at low temperature.

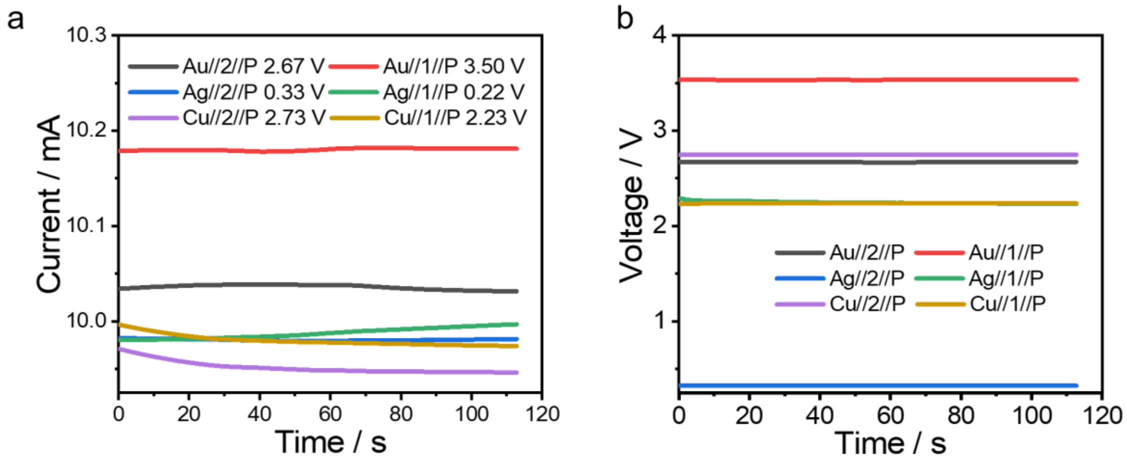

Supplementary Figure 8. Stability upon conduction of electricity of hybrid crystals over prolonged periods of time. (a) Plot of current versus time after applying a constant voltage to Au, Ag, Cu //1,2//P. (b) Plot of voltage versus time after applying a constant current to Au, Ag, Cu //1,2//P.

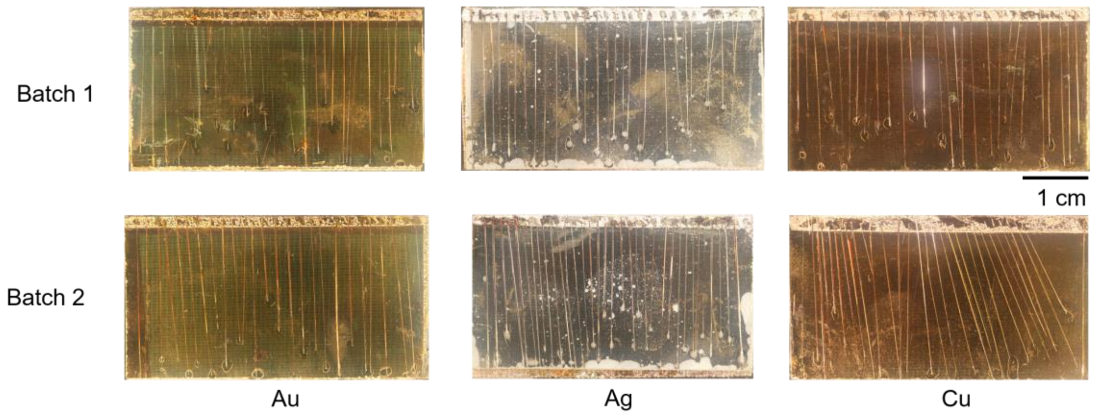

Supplementary Figure 9. Photographs of hybrid organic crystals. Optical photographs of Au,Ag,Cu//1,2 prepared from different batches.

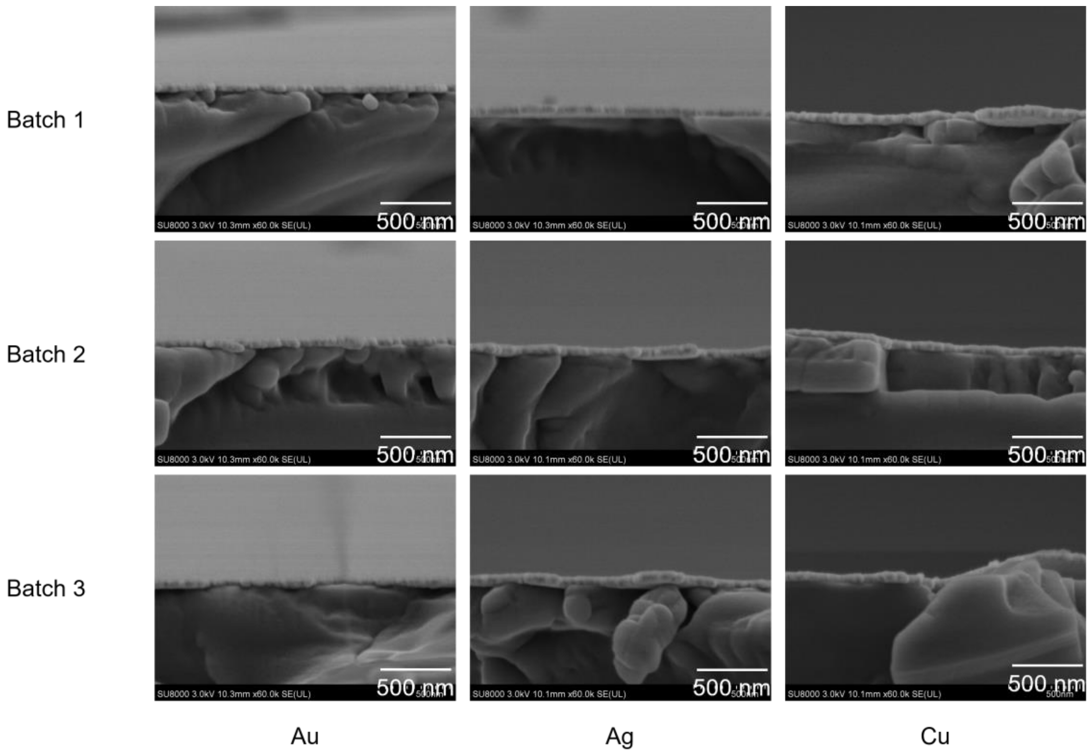

Supplementary Figure 10. SEM testing of hybrid organic crystals. SEM images of the cross-sections of Au,Ag,Cu//2 prepared from different batches.

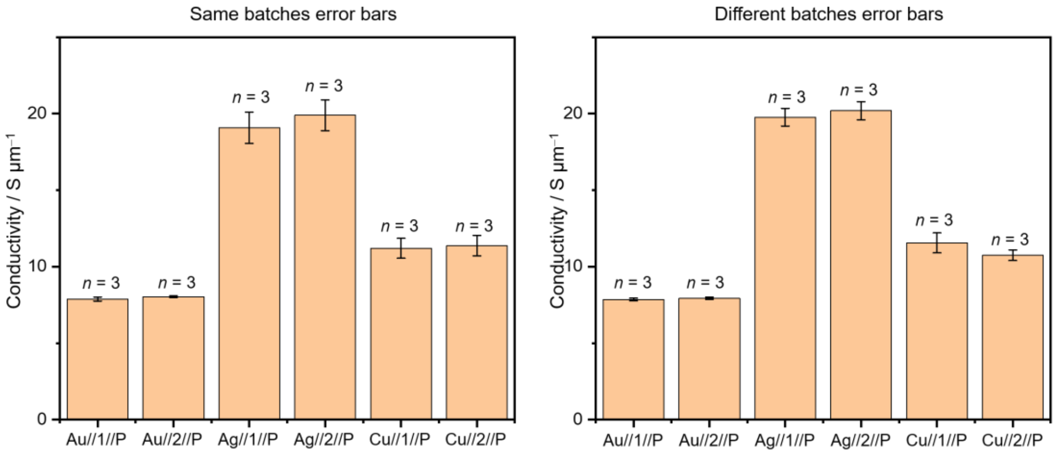

Supplementary Figure 11. Conductivity measured from multiple crystals of the same composition. The error bars represent the standard deviations from multiple crystals. The number of samples (n) for each batch is also given.

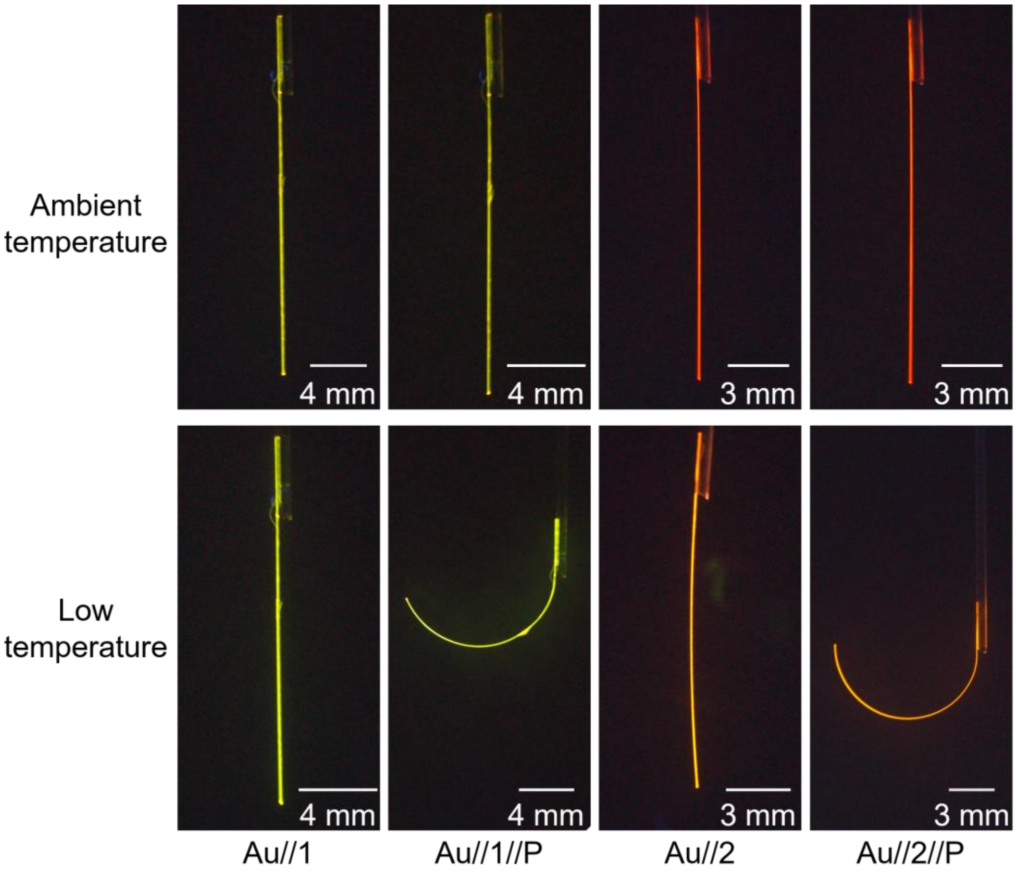

Supplementary Figure 12. Photographs of hybrid organic crystals at different temperatures. Optical images of Au//1, Au//1//P, Au//2, and Au//2//P recorded at ambient temperature and at low temperature.

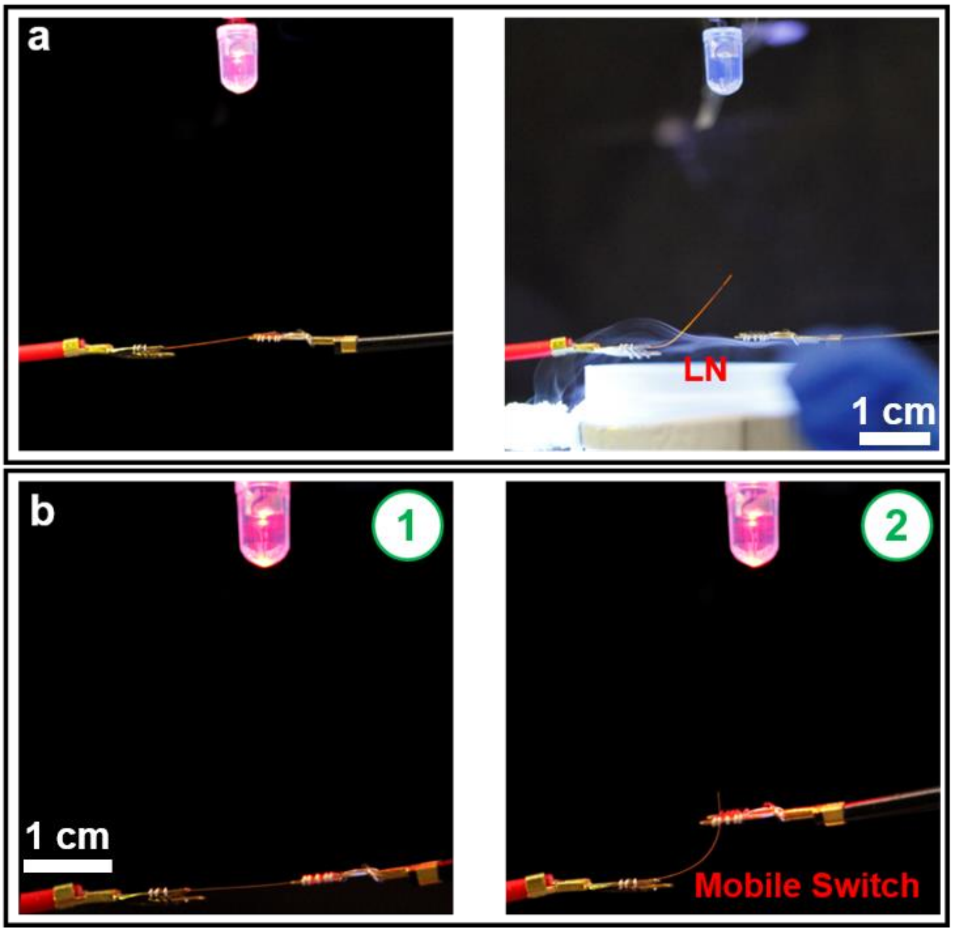

Supplementary Figure 13. Optical images of the experimental setup. (a) Optical images of hybrid organic crystal temperature switching device. (b) Photograph of a current switching device at a specific temperature by adjusting the position (horizontal or height) of the contacts (conductive plates). (The number one represents the lowering conductive plate to monitor higher temperatures. The number two represents the elevated of the conductive plate to monitor the lower temperature)

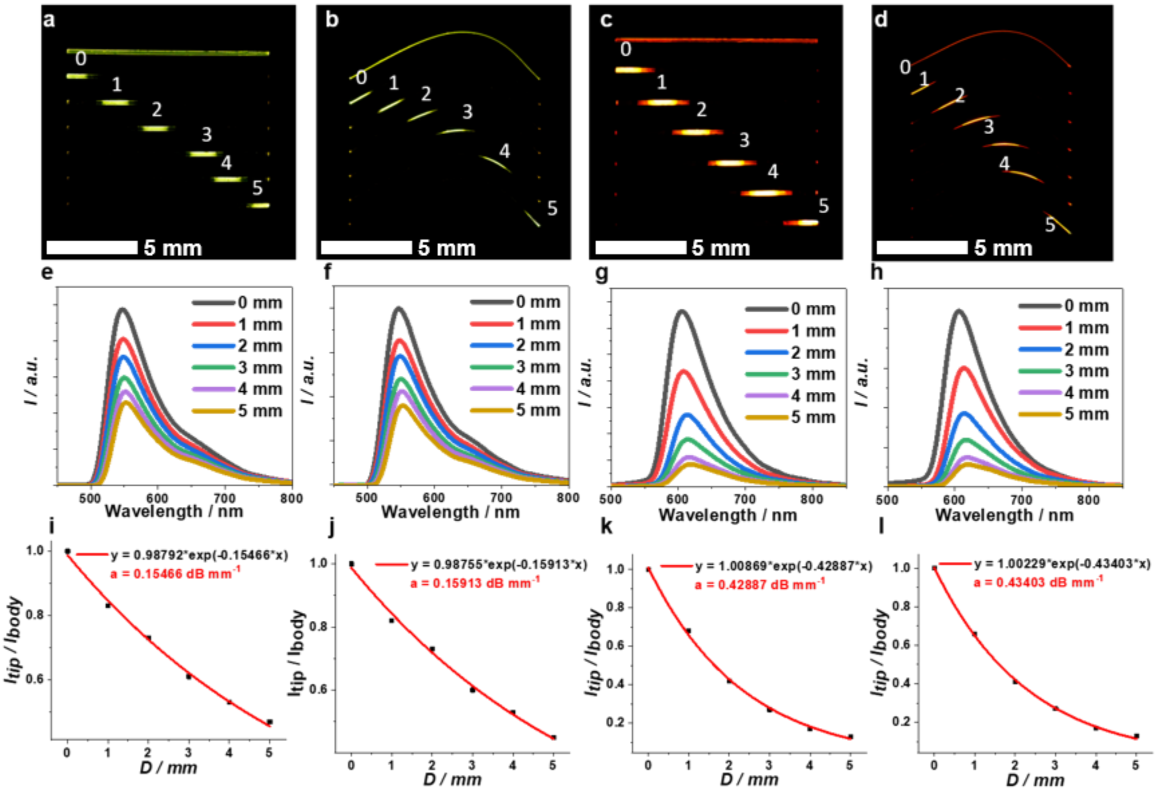

Supplementary Figure 14. Waveguide capability of the hybrid organic crystals. (a–d) Image of Au//1,2//P used as a waveguide, (a) Au//1//P in straight state, (b) Au//1//P. in bent state, (c) Au//2//P in straight state, (d) Au//2//P in bent state. (e–h) Fluorescence spectra were collected at the fixed end of the crystal, while the crystals were exited at different position by 355 nm laser, position differences between the fixed end and the excitation position is defined as distance (mm). The panels e, f, g, and h are corresponding to the crystals shown in panels a, b, c, and d, respectively. (i–l) Decay of intensity with distance I_{tip}/I_{body}. The optical loss coefficient (α) is obtained by a single exponential fitting function. $I_{tip}/I_{body} = A\exp(-\alpha D)$, in which I_{tip} and I_{body} are the fluorescence intensities measured at the fixed end and the excitation position, respectively. A is the optical loss coefficient and D is position differences between the fixed end and the excitation position. (i) Au//1//P. in straight state, (j) Au//1//P. in bent state, (k) Au//2//P. in straight state, (k) Au//2//P. in bent state.

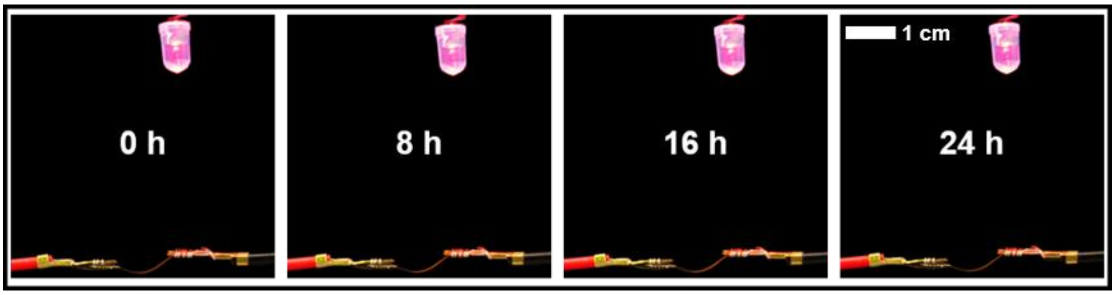

Supplementary Figure 15. Optical images of hybrid organic crystals at different times. Optical images of Au//2//P connected to circuit for 0, 8, 16 and 24 hours.

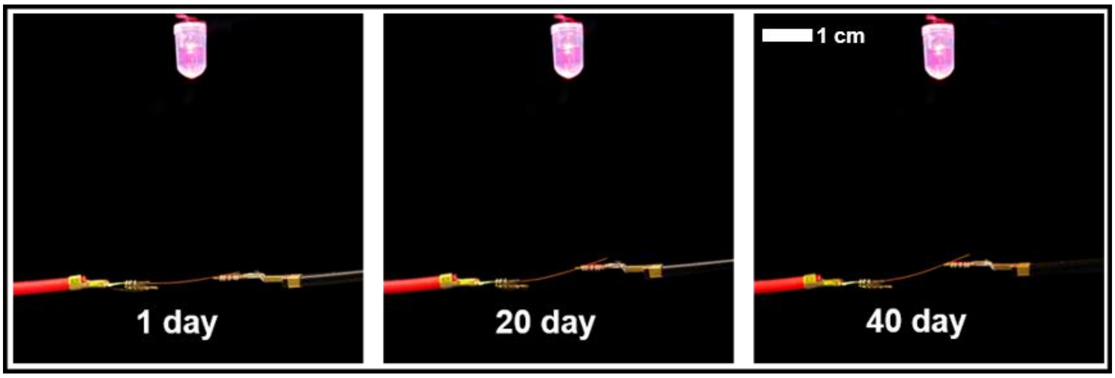

Supplementary Figure 16. Optical images of hybrid organic crystals at different times. Optical images of Au//2//P connected to circuit for 1, 20 and 40 days.

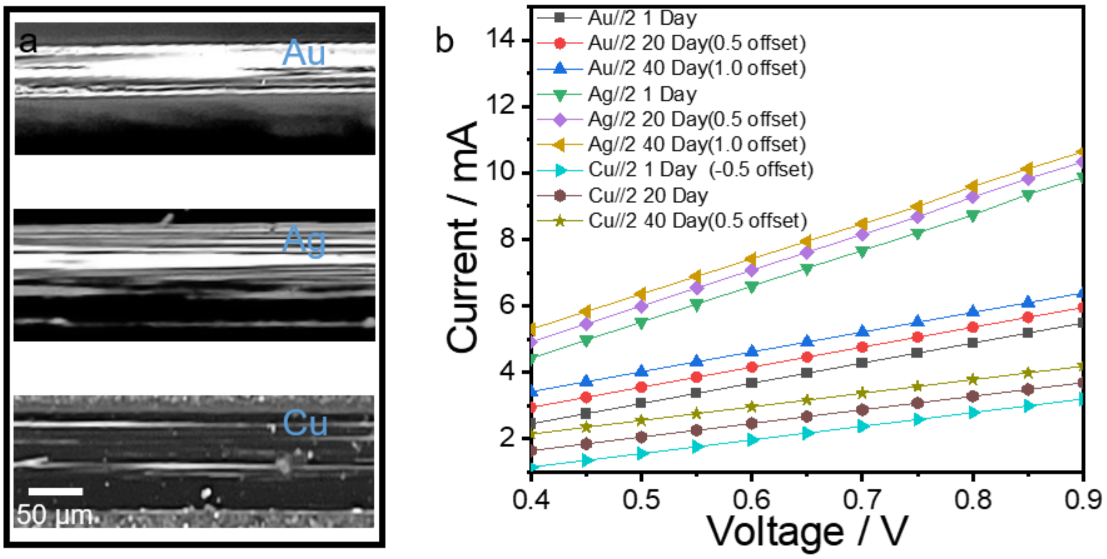

Supplementary Figure 17. Conductivity testing of hybrid organic crystals. (a) SEM images of M//2 (top view). (b) *I*–*V* plot of M//2 for 1, 20 and 40 days. Vertical offsets have been applied for clarity.

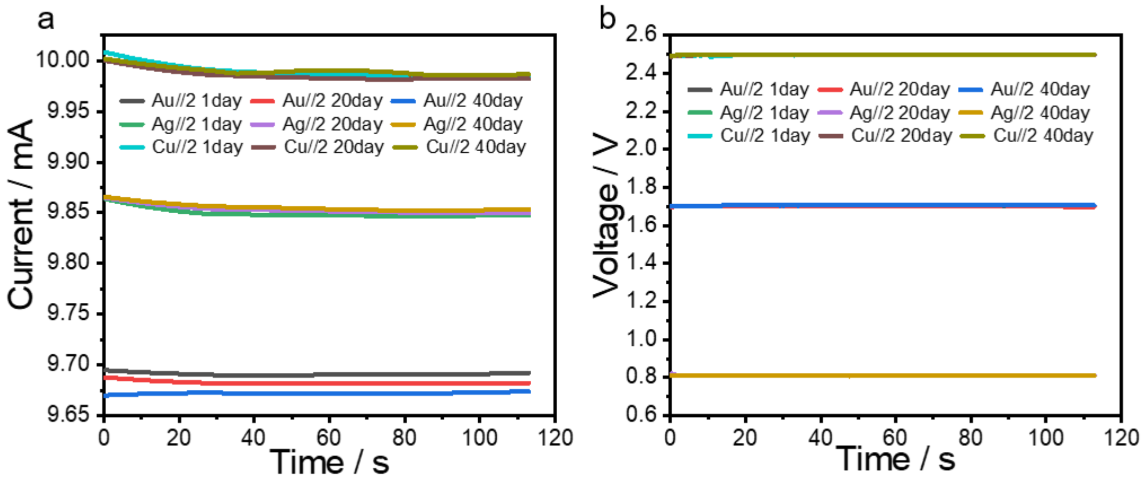

Supplementary Figure 18. Conductivity duribility test of the hybrid organic crystals. (a) Plot of current versus time after applying a constant voltage to M//2 for 1, 20 and 40 days. (b) Plot of voltage versus time after applying a constant current to M//2 for 1, 20 and 40 days.

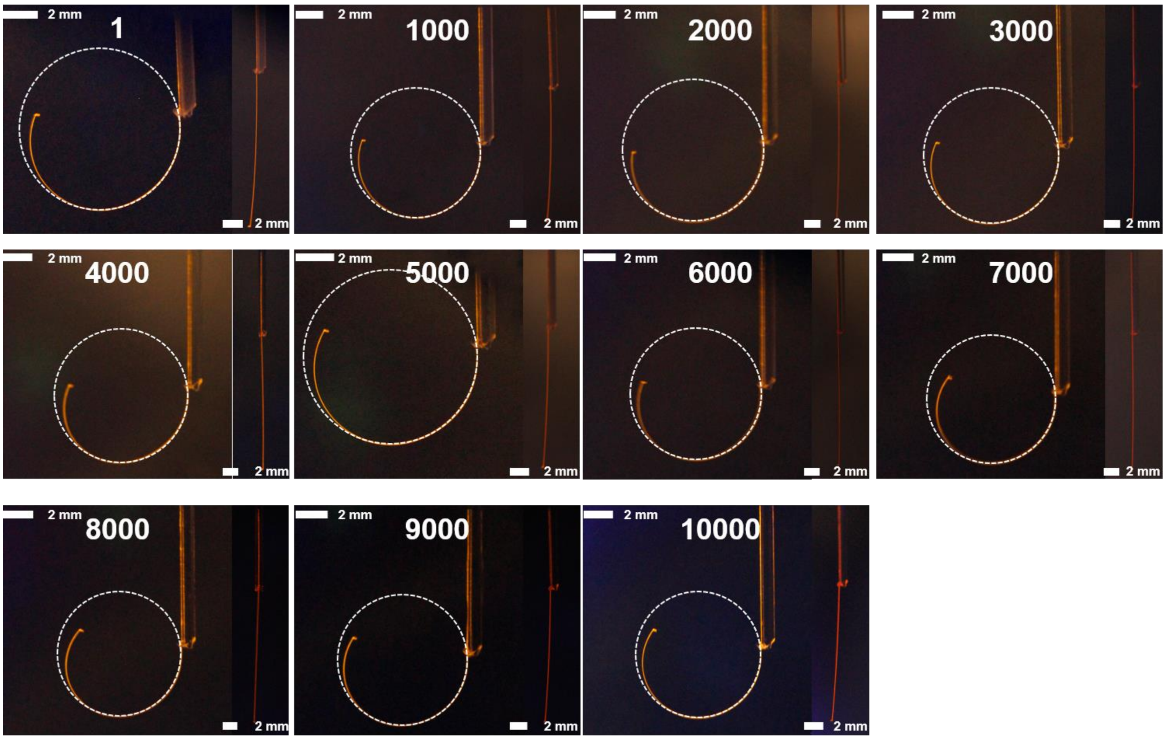

Supplementary Figure 19. Durability testing of hybrid organic crystals. Optical images of bending durability tests for Au//2//P.

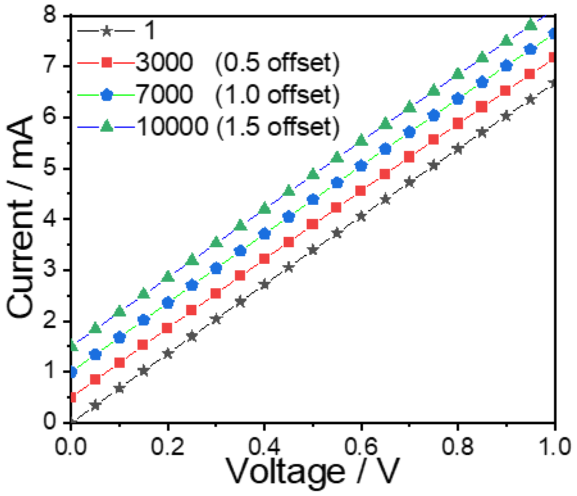

Supplementary Figure 20. Conductivity duribility test of the hybrid organic crystals. *I*–*V* plot of Au//2//P Au after different bending cycles. Curves are plotted with y offsets for clarity reason.

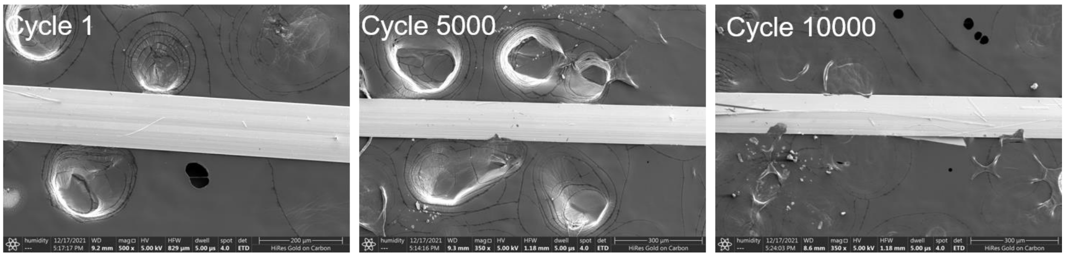

Supplementary Figure 21. The SEM testing of hybrid organic crystals. SEM images of Au//2//P with different number of bending circles.

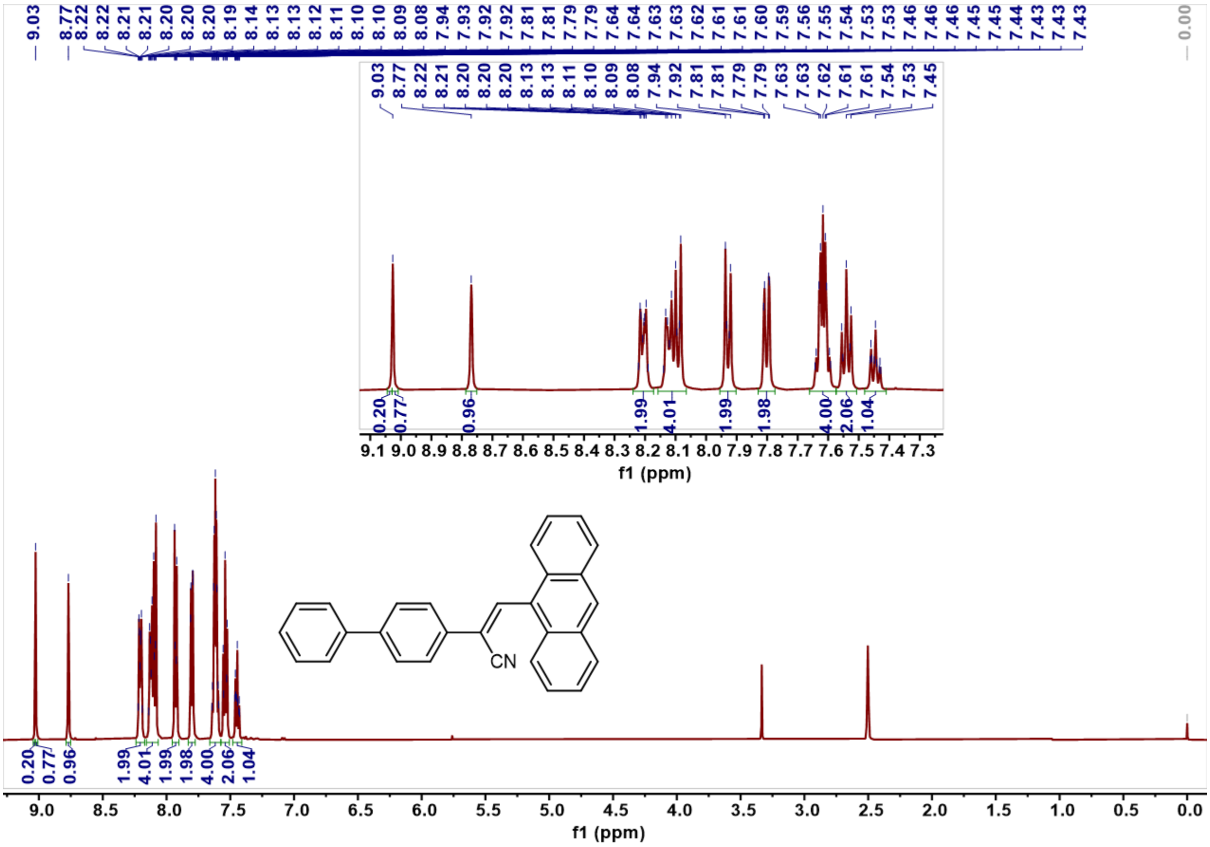

Supplementary Figure 22.^{1}H NMR spectrum of compound 1 (Chloroform-*d*, 500 MHz).

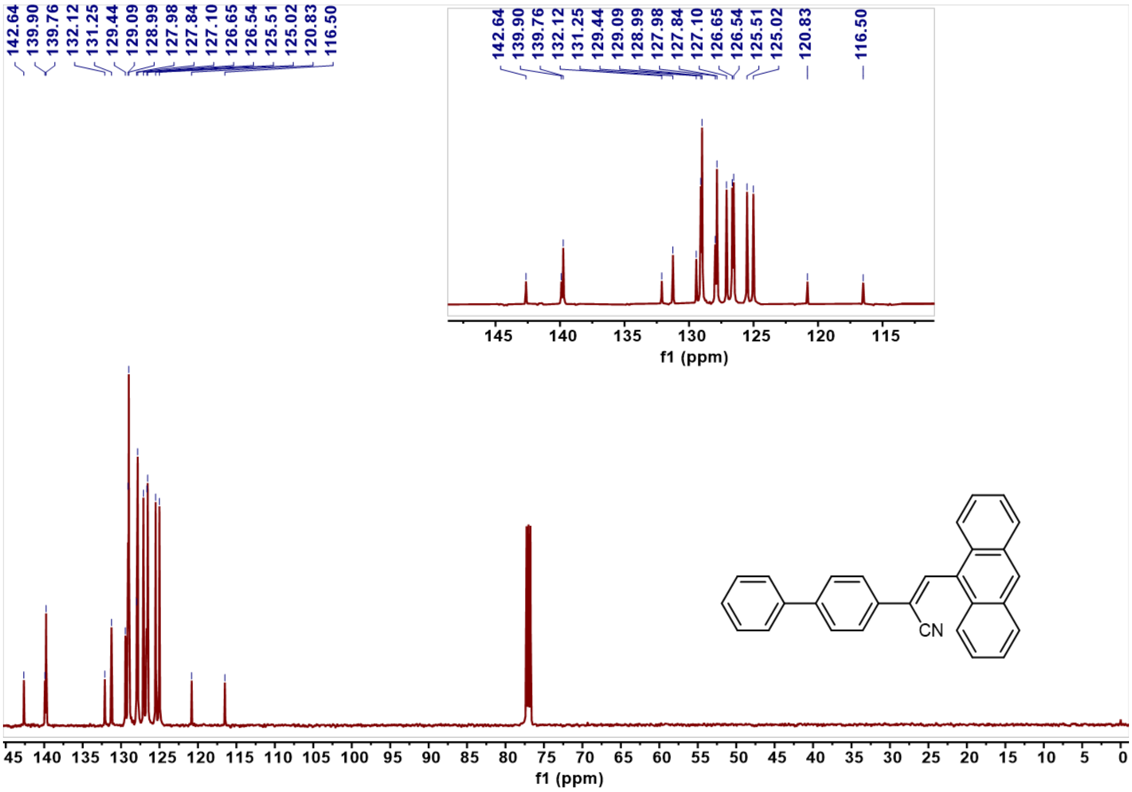

Supplementary Figure 23. ^{13}C{1H} NMR spectrum of compound 1 (Chloroform-*d*, 126 MHz).

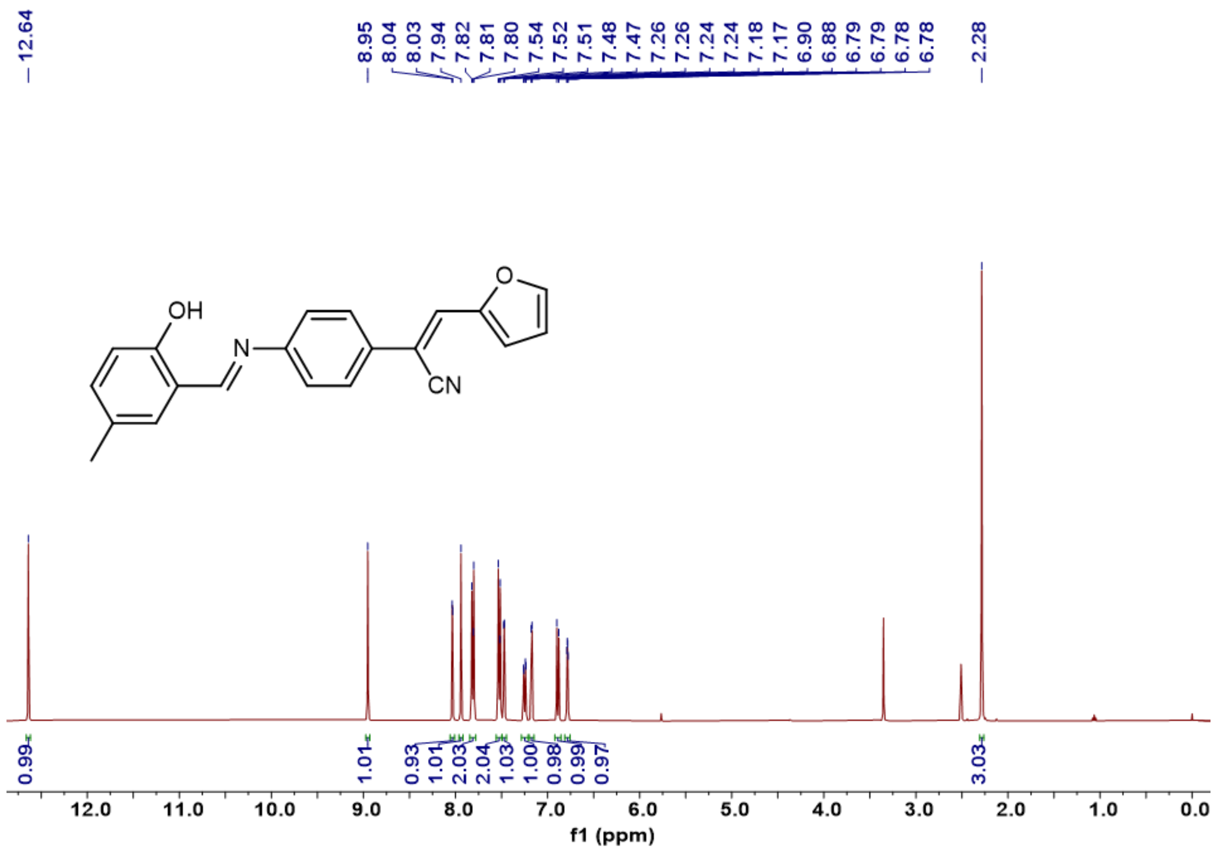

Supplementary Figure 24.^{1}H NMR spectrum of compound 2 (DMSO-d_6, 400 MHz).

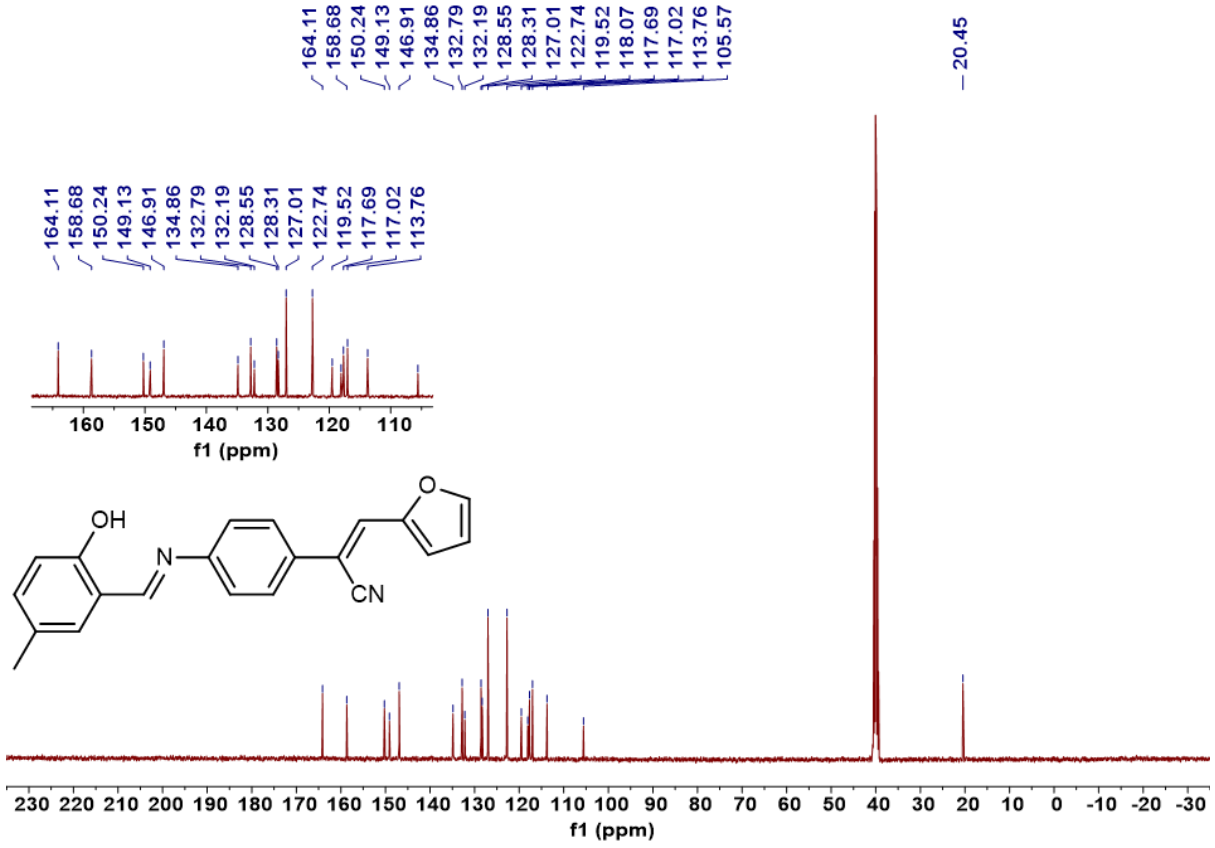

Supplementary Figure 25. ^{13}C{1H} NMR spectrum of compound 2 (DMSO-d_6, 101 MHz).

Supplementary tables

Supplementary Table 1. Lengths, widths, and thicknesses of crystals of 1 and 2

Size table	1	2
Length	1–3 cm	1–3 cm
Width	50–300 µm	50–220 µm
Thickness	20–130 µm	10–22 µm

Supplementary Table 2. Conductivity of the hybrid crystals with various composition in different conditions

Name	Width / µm	Length / mm	Thickness / nm	Conductivity / (S μm^{-1})
Au//2//P	52.67	1.50	80	8.080976
Au//1//P	135.14	2.10	80	7.88214
Ag//2//P	135.00	0.80	80	20.93947
Ag//1//P	85.24	3.00	80	18.83375
Cu//2//P	98.70	2.30	80	10.89802
Cu//1//P	93.20	1.50	80	11.7296
Au//2//P 20 °C	65.89	1.65	80	7.9468
Au//2//P –160 °C	66.89	1.65	80	9.021278
Au//2//P 0°	163.59	1.60	80	8.108416
Au//2//P 30°	163.59	1.60	80	8.086514
Au//2//P 60°	163.59	1.60	80	8.082744
Au//2//P 90°	163.59	1.60	80	8.075953
Au//2//P 120°	163.59	1.60	80	8.067304
Au//2//P 150°	163.59	1.60	80	8.058671
Au//2//P 180°	163.59	1.60	80	8.049688

Supplementary Table 3. Reproducibility of the conductivity measurements of the crystals

Batches	Name	Width / µm	Length / mm	Thickness / nm	Conductivity / (S µm^{-1})
1	Au//1//P	112.37	1.00	80	7.795593
1	Au//1//P	87.76	0.50	80	8.034112
1	Au//1//P	58.02	0.80	80	7.838520
2	Au//1//P	70.69	1.30	80	7.959925
3	Au//1//P	60.53	1.20	80	7.812410
1	Au//2//P	53.21	1.30	80	8.024696
1	Au//2//P	62.10	0.80	80	7.988232
1	Au//2//P	55.73	2.10	80	8.093614
2	Au//2//P	54.20	0.60	80	7.936724
3	Au//2//P	106.28	1.00	80	7.865398
1	Ag//1//P	104.22	1.20	80	20.241345
1	Ag//1//P	141.19	1.60	80	18.322393
1	Ag//1//P	106.28	1.00	80	18.680604
2	Ag//1//P	120.61	1.20	80	19.932765
3	Ag//1//P	58.95	1.10	80	19.129725
1	Ag//2//P	197.17	1.80	80	20.799086
1	Ag//2//P	53.26	1.00	80	20.070580
1	Ag//2//P	98.72	0.90	80	18.815597
2	Ag//2//P	59.34	0.60	80	19.623527
3	Ag//2//P	62.13	1.00	80	20.167421
1	Cu//1//P	139.49	1.00	80	11.812360
1	Cu//1//P	167.53	1.00	80	10.515717
1	Cu//1//P	77.28	0.60	80	11.288907
2	Cu//1//P	190.27	1.10	80	12.044161
3	Cu//1//P	96.31	0.90	80	10.818833
1	Cu//2//P	126.15	1.00	80	10.616285
1	Cu//2//P	113.77	1.00	80	11.641258
1	Cu//2//P	86.24	0.90	80	11.867402
2	Cu//2//P	109.21	1.10	80	11.137885
3	Cu//2//P	56.95	0.60	80	10.492348

Supplementary Table 4. Calculated data related to crystals in different states

Name	Width / μm	Length / mm	Thickness / nm	Conductivity / (S μm^{-1})
Time 1	153.59	1.50	80	8.154755
Time1000	153.59	1.50	80	8.150442
Time 2000	153.59	1.50	80	8.145797
Time 3000	153.59	1.50	80	8.144271
Time 4000	153.59	1.50	80	8.141532
Time 5000	153.59	1.50	80	8.137865
Time 6000	153.59	1.50	80	8.128786
Time 7000	153.59	1.50	80	8.126078
Time 8000	153.59	1.50	80	8.122245
Time 9000	153.59	1.50	80	8.116837
Time 10000	153.59	1.50	80	8.091105
Au//2 1day	119.24	1.20	80	7.659166
Au//2 20day	119.24	1.20	80	7.599765
Au//2 40day	119.24	1.20	80	7.515664
Ag//2 1day	127.85	1.90	80	20.21947
Ag//2 20day	127.85	1.90	80	20.1947
Ag//2 40day	127.85	1.90	80	20.1549
Cu//2 1day	82.10	2.00	80	12.51056
Cu//2 20day	82.10	2.00	80	12.48615
Cu//2 40day	82.10	2.00	80	12.47867

Supplementary Table 5. Crystallographic data of crystal 1

Compound	1
Formula	$C_{29}H_{19}N$
Temperature / K	100.0
Formula Weight	381.45
Crystal System	orthorhombic
Space Group	*Fdd2*
a / Å	6.9427(15)
b / Å	38.717(3)
c / Å	5.5033(2)
α / °	90
β / °	90
γ / °	90
Volume / Å^3	7871.3(7)
Z	16
Density / (g cm^{-3})	1.288
μ /mm^{-1}	0.074

F_{000}	3200.0
Reflections collected	53757
Independent reflections	5508
CCDC No.	2157104
