# Supplementary material for: Electrically conductive hybrid organic crystals as flexible optical waveguides
Source: Nat Commun. 2022 Dec 22;13:7874. doi: 10.1038/s41467-022-35432-w (PMC9780324; doi:10.1038/s41467-022-35432-w)
Supplement: Supplementary file 2 — Description of Additional Supplementary Files [file 41467_2022_35432_MOESM2_ESM.pdf]

Description of Additional Supplementary Files

File Name: Supplementary Movie 1
Description: Video recording showing a crystal of compound 3 that becomes brittle and disintegrates at low temperature.

File Name: Supplementary Movie 2
Description: Video recording showing a hybrid crystal of compound 3 coated with polymer that disintegrates at low temperature.

File Name: Supplementary Movie 3
Description: Rapid curling of a hybrid crystal Au//2//P when placed over liquid nitrogen (the temperature of the cooled crystal is about −160 °C).
